# Dietary effect on glucose homeostasis is modulated by a loss-of-function variant in the sucrase-isomaltase gene: a randomised, dietary crossover intervention in Inuit

**DOI:** 10.1007/s00125-026-06723-4

**Published:** 2026-04-17

**Authors:** Ninna Senftleber, Marie Mathilde B. Christensen, Bendix Carstensen, Frederik Filip Stæger, Michael B. Frøst, Matthew P. Gillum, Torben Hansen, Marit E. Jørgensen

**Affiliations:** 1 Steno Diabetes Center Greenland, Nuuk, Greenland; 2 Clinical and Translational Research, Copenhagen University Hospital, Steno Diabetes Center Copenhagen, Herlev, Denmark; 3 Department of Biology, Faculty of Science, University of Copenhagen, Copenhagen N, Denmark; 4 Department of Food Science, University of Copenhagen, Frederiksberg, Denmark; 5 Department of Biomedical Sciences, University of Copenhagen, Copenhagen, Denmark; 6 Novo Nordisk Foundation Center for Basic Metabolic Research, Faculty of Health and Medical Sciences, University of Copenhagen, Copenhagen, Denmark; 7 University of Southern Denmark, National Institute of Public Health, Copenhagen, Denmark

**Keywords:** Congenital sucrase-isomaltase deficiency, Dietary intervention, Gene × diet interaction, Glucose homeostasis, Greenland, Inuit, Loss-of-function variant, Randomised crossover trial, Sucrase-isomaltase, Sucrose

## Abstract

**Aims/hypothesis:**

Homozygous carriers of a loss-of-function variant in the sucrase-isomaltase (SI) gene (c.273_274delAG) are unable to digest sucrose and parts of starch. The variant is common only in Indigenous Arctic populations such as the Greenlandic Inuit and has been associated with a healthier metabolic profile. In a unique gene–diet intervention, we aimed to study whether the *SI* genotype modulates the effect of two different diets on glucose homeostasis and lipids.

**Methods:**

A genotype-based randomised crossover trial was conducted in homozygous *SI* carriers and non-carriers in Nuuk and Maniitsoq (Greenland), with two 3 day interventions and a 7 day wash-out period. Participants were ≥18 years, had no gastrointestinal disorders, diabetes nor carried an Inuit-specific high-risk type 2 diabetes variant in *TBC1D4*. The interventions were as follows: a Greenlandic fish- and meat-rich diet; and an isoenergetic Western diet with 11% energy from sucrose. The order of the diets was randomised by the participants using a dice and participants and personnel were not blinded. The primary outcome was glucose variability measured as CV. Fasting blood samples were drawn before and after each intervention for measurement of lipids, insulin and C-reactive protein. We assessed genotype × diet interaction effects using linear mixed models. The study was reported in accordance with the CONSORT 2010 statement: extension to randomised crossover trials and the consolidated criteria for strengthening reporting of health research involving Indigenous peoples (the CONSIDER statement).

**Results:**

Seventeen carriers and 16 non-carriers completed the intervention. CV was higher on the Western diet than on the Greenlandic diet for non-carriers (β=5.23% [95% CI 3.02, 7.45]) but not for the carriers (β 1.27% [−0.86, 3.4]). Carriers had a 20% lower CV on the Western diet compared with non-carriers (*p*_genotype×diet_=0.015). Carriers had lower fasting insulin levels than non-carriers at baseline, and the Greenlandic diet decreased the levels in non-carriers by 22.7 pmol/l (95% CI 5.1, 40.3) but not in carriers (*p*_genotype×diet_=0.009). We observed no *SI ×* diet interaction effects on lipids levels.

**Conclusions/interpretation:**

Homozygous loss-of-function *SI* carriers show better glycaemic management than non-carriers on a Western diet, suggesting SI inhibition as a potential treatment target.

**Trial registration:**

ClinicalTrials.gov NCT05375656

**Funding:**

Independent Research Fund Denmark, Greenland Research Council and Brugseni.

**Graphical Abstract:**

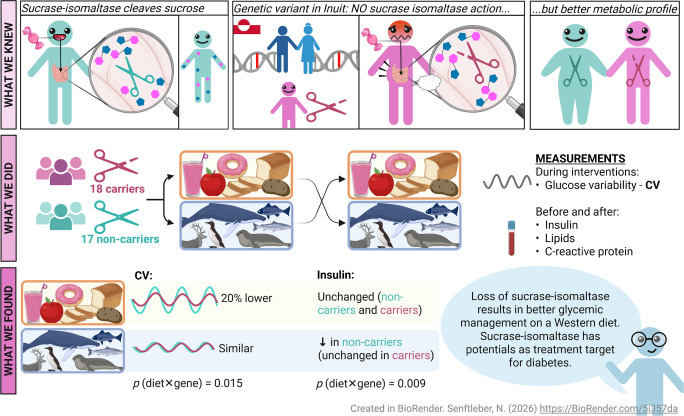

**Supplementary Information:**

The online version of this article (10.1007/s00125-026-06723-4) contains peer-reviewed but unedited supplementary material.


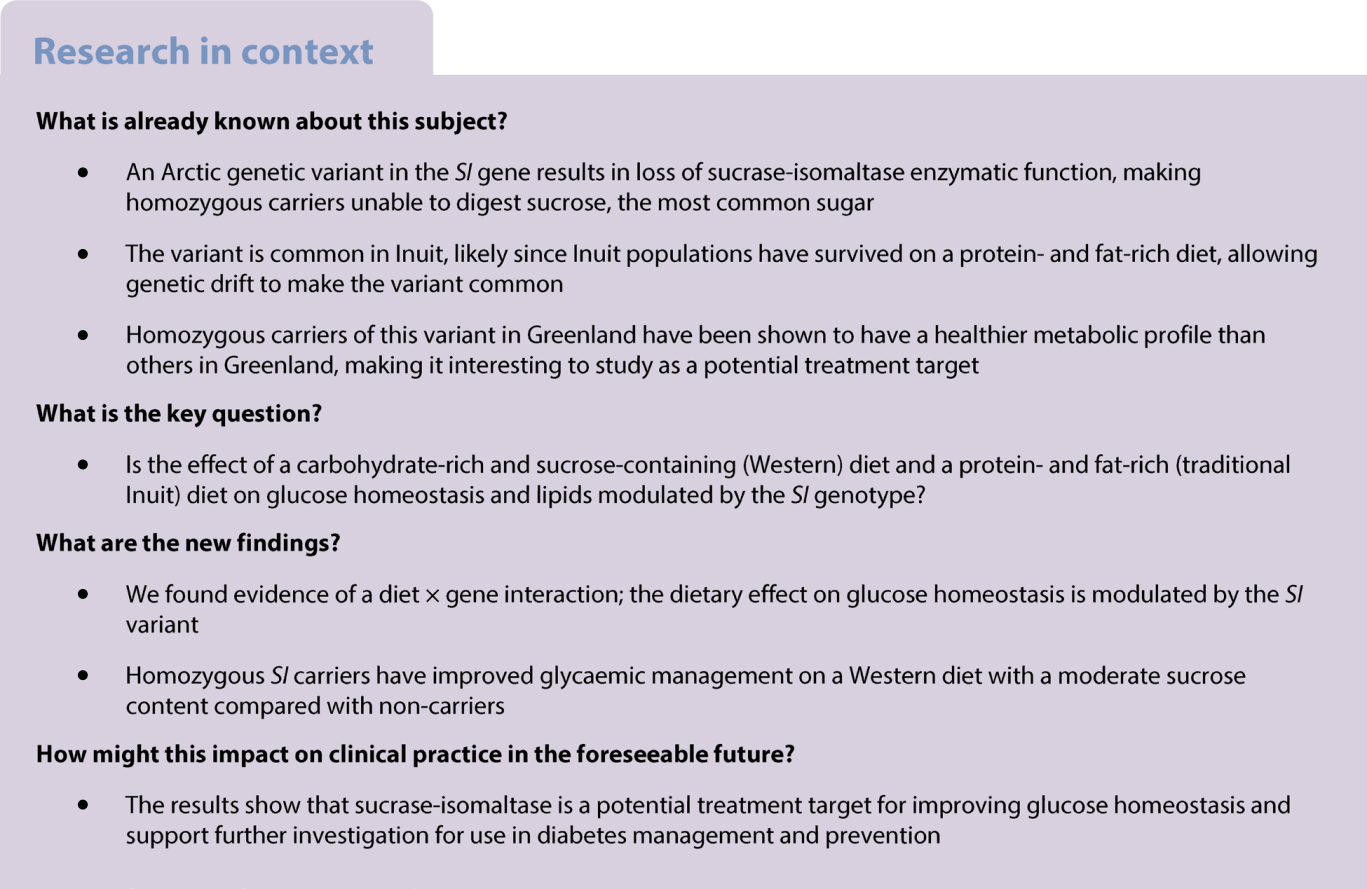


## Introduction

Greenland is located in the Arctic and has a population of only 56,000 [1]. The vast majority of the population is of Inuit ancestry [2] and has experienced a dramatic increase in the burden of obesity and metabolic diseases during the past few decades [3]. Before the 1980s, type 2 diabetes was rare in Greenland [4], while 9% of the population was reported to have type 2 diabetes in a large population survey conducted in 2005–2010 [5]. However, approximately 2% of the Greenlandic population are homozygous (HO) carriers of a unique Arctic variant in the *SI* gene (c.273_274delAG) predicted to result in complete loss of sucrase-isomaltase (SI) enzyme function [6], and which seems to have metabolically beneficial effects [7]. With an allele frequency of 14.2% in Greenland and 20% in the Inuit ancestry genome [7], the variant is common and the prevalence of congenital SI deficiency is many-fold higher than in other populations [8]. The SI enzyme, located on the intestinal brush border membrane, breaks down sucrose and starch components (major dietary components in most populations) into absorbable monosaccharides [9–11]. Thus, HO carriers of the *SI* variant cannot digest these nutrients, causing symptoms such as pain, bloating and diarrhoea after sucrose intake [6, 12, 13]. Interestingly, HO carriers of the variant have shown a healthier metabolic profile, including lower BMI, fat percentage, body weight and lipid levels, being unexplained by their reduced sucrose intake. A knockout mouse model supported this phenotype, and Greenlandic HO carriers furthermore showed indications of having a lower risk of some cardiovascular diseases [7]. Heterozygous carriers might have partial SI function, though clinical data remain limited [14].

Although the *SI* variant is very rare in non-Arctic populations and common in Arctic populations, there is no signs of positive selection [7]. Historically, the Inuit depended on a diet rich in protein and fat from marine and land animals, with minimal carbohydrates [15, 16]. Thus, the *SI* variant likely became prevalent through genetic drift and lack of negative selection, since it likely did not affect the chance of survival on the low-carbohydrate diet. However, the intake of imported foods high in sucrose and carbohydrates has increased during the past decades [16]. Diets high in sugars and fast-digesting carbohydrates have been associated with risk of type 2 diabetes [17, 18], and some hypothesise that the dietary transition is partly responsible for the increasing burden of metabolic diseases in the Indigenous Arctic populations, though studies have shown conflicting results [19–21].

The high prevalence in Greenland of a variant causing complete loss of SI function provides a unique opportunity to study the consequences of SI deficiency in humans. In this randomised crossover trial, we investigated whether the *SI* genotype modifies the effect of a Western diet and a traditional Greenlandic diet on glycaemic variability. We hypothesised that HO carriers of the variant have a lower glycaemic variability on a Western diet than non-carriers but similar variability on a traditional Greenlandic diet, suggesting a metabolic advantage being a carrier when consuming a Western diet [22–25]. Secondary outcomes included lipid, insulin and C-reactive protein levels. We hypothesised that lack of SI enzymatic activity is a metabolic advantage in a modern, Western society, where food is not limited and the diet is high in starch and sucrose, thus making SI a potential treatment target for improving metabolic health.

## Methods

Participants were given information written and orally in Greenlandic or Danish, depending on their preference, and participants gave informed consent for analysis of collected data and samples, including data not covered in this paper. The full protocol of the intervention has been published elsewhere, including details on secondary aims not reported in this paper [26]. The Ethics Committee of Greenland approved the study (KVUG 2021-19). The study was reported in accordance with the CONSORT 2010 statement: extension to randomised crossover trials [27] and the Consolidated criteria for strengthening reporting of health research involving Indigenous peoples: the CONSIDER statement [28] (electronic supplementary material [ESM] Table 1).

### Participants and recruitment

We recruited participants aged ≥18 years from Maniitsoq and Nuuk (ESM Fig. 1) by inviting HO *SI* carriers and non-carriers identified in previous population surveys [7]. We invited all known carriers at the two study sites, irrespective of sex or gender. A local, Greenlandic research assistant at each location was responsible for recruitment and overall communication with participants throughout the study, and was a pivotal part of the data collection. In Nuuk, we recruited six additional HO *SI* carriers via screening individuals experiencing symptoms of sucrose malabsorption for inclusion by performing an oral sucrose tolerance test after an overnight fast to confirm lack of sucrose digestion. Included individuals gave consent for later whole-genome sequencing, including confirmation of HO *SI* carrier status. Participants recruited from the population survey completed the oral sucrose tolerance test at the end of the study, to confirm the inability or ability to digest sucrose. Heterozygous carriers were not included in the study, as the impact of the *SI* variant on the enzymatic activity is still not known in these individuals and the healthier metabolic profile has only been observed in HO carriers [7].

We recruited HO carriers and non-carriers in a 1:1 ratio. To achieve two groups with similar demographics, each non-carrier was recruited to roughly match the HO carriers on sex, age and BMI at the time of the population survey, and Inuit/European genetic admixture. The list of potential non-carriers matching each carrier on the selected characteristics was generated by a nearest-neighbour matching approach using propensity scores with the R package MatchIt [29]. Sex was determined by the personal identification number.

We applied the following inclusion criteria:

- HO group: HO carriers of the *SI* loss-of-function variant
- HO group: <10% increase in blood glucose in an oral sucrose tolerance test
- Non-carrier group: non-carriers of the *SI* loss-of-function variant
- Non-carrier group: >10% increase in blood glucose in an oral sucrose tolerance test

Exclusion criteria for both groups were as follows:

- Severe gastrointestinal disorders (e.g. inflammatory bowel disease, gastrointestinal cancer and ulcer)
- Known diabetes
- HO carriers of the Inuit-specific *TBC1D4* variant, a type 2 diabetes high-risk variant [30]

If a non-carrier dropped out of the study, we recruited a new non-carrier when possible, matching the HO carrier of the original match. As all known HO carriers at the two study sites were invited to participate, as well as all screening-detected HO individuals, it was not possible to recruit new HO individuals to replace dropouts.

### Study design

The study was performed as a randomised, crossover dietary intervention. The participants followed each of the diets for 3 days with a wash-out period of 7 days in between. Randomisation of which order the participants received the two diets removed the risk of a systematic carry-over effect of the first diet and was performed on a demographically matching pair of one HO carrier and one non-carrier to achieve two similar groups, as the sample size was relatively small. This was done by one participant of the matching pair throwing a dice and the pair of participants were subsequently allocated. However, due to a procedural error, one participant received the diets in an order opposite to that assigned to the matched pair (the participant withdrew halfway through the study). The study was not blinded due to the nature of the intervention. The trial was conducted in Maniitsoq and Nuuk in January–May 2022 and no methodological changes were applied after trial commencement.

### User study

To plan an intervention that would be acceptable, we conducted a user-involvement study beforehand. Carriers and non-carriers were interviewed about their experiences with eating sucrose- and starch-containing foods and, importantly, about their opinion on research on diet and genetics in Greenland in general. The primary interviewer was Greenlandic and conducted the interview in the language preferred by the participants. The results from the user study have been published elsewhere [13].

### Intervention

The Western diet was high in starch, mostly from bread and pasta. Sources of sucrose were fruit yoghurt, fruits, orange juice and biscuits. Dinner meals were frozen convenience meals supplied with extra vegetables. The diet had the following nutrient composition (expressed as percentage of total energy derived from each macronutrient [i.e. energy % (E%)]):

- Sucrose 11 E%
- Other carbohydrates 47–49 E%
- Protein 14 E%
- Fat 25 E%

The traditional Greenlandic diet was high in protein and fat from raw and dried fish, reindeer meat, dried whale meat and eggs. Very few carbohydrate-containing foods were provided. The majority of the foods were provided frozen and participants prepared dinner meals themselves. The nutrient composition was:

- Sucrose 0 E%
- Other carbohydrate 5 E%
- Protein 36 E%
- Fat 57 E%

The two diets were isoenergetic and, in order to increase compliance, we provided each participant with 34,500 kJ/day (8246 kcal/day), being enough to cover the requirement of the entire household. Moreover, they received a dietary chart with instructions for every meal and snacks/light meals; they were instructed to consume enough food to feel satiated and to mark in the chart which foods they consumed and if they consumed other foods than those provided, though this was advised against. The charts were used as dietary control and to evaluate compliance in the groups using information on both the intakes of the intervention foods as well as other foods and beverages consumed. For most foods, information on consumption was expressed as a frequency (e.g. number of times per day that dried fish was consumed) and, for some foods, as consumed number of the food unit (e.g. number of fruits per day). All dairy products were lactose-free.

### Outcomes and measurements

ESM Fig. 2 provides an overview of the study timeline, outcomes and sampling.

### Glycaemic variability (primary outcome)

During the interventions and wash-out period, participants had their blood glucose monitored using the 14 day Freestyle Libre Pro system and were blinded to the measurements. The device was inserted on the back of the upper left or right arm and was replaced as soon as possible if it became detached. Glycaemic variability was measured as CV, estimated as SD/mean (glucose) × 100% during each intervention period, and served as the primary outcome [31]. We also assessed mean amplitude of glucose excursion (MAGE) and SD.

### Blood samples after an overnight fast

Blood samples were drawn before and after each intervention (i.e. at four visits), after an overnight fast, for biochemical analyses of serum insulin, plasma C-reactive protein, triacylglycerol, VLDL-, LDL- and HDL-cholesterol, and total and remnant cholesterol. HbA_1c_ and plasma glucose were measured only at baseline. Blood samples were shipped to Denmark for analyses and will be destroyed when these are complete. Blood samples collected for potential future analyses are stored in Nuuk, Greenland. Other details regarding sampling and analyses have been described previously [26].

### Anthropometric assessment

Weight and bioimpedance were measured on a leg-to-leg Tanita Body Composition Analyser (SC-330; Tanita Corporation, Tokyo, Japan). Height and waist and hip circumference were measured to the nearest 0.5 cm. For these measurements, the participants wore light underwear and the same devices were used on all participants.

### Genetic ancestry

Six of the 38 participants were not part of the public health surveys. These were whole-genome sequenced and merged into the genetic dataset from the public health surveys, resulting in a total of 6002 individuals. Sites with minor allele frequency >5% and missingness <1% were LD-pruned using PLINK (v1.90b7) [32] in 5 MB windows, step-size of 1 and R2 threshold of 0.5 resulting in 335,332 sites after filtering. Admixture proportions were estimated using the ADMIXTURE software (v.1.3.0) [33], assuming two ancestral populations and with five different start seeds. All five seeds resulted in identical loglikelihoods, indicating that the admixture model had converged. Finally, the admixture proportions of the 38 participants were extracted. The merging was done to assure proper modelling of the two ancestries, which requires unadmixed individuals of each ancestry.

### Data management and statistical analyses

Before recruiting participants, we planned MAGE to be the measure of the primary outcome, hence the sample size calculation was based on 80% power to detect a difference of 0.5 mmol/l in MAGE between the genotypes on a Western diet when using a 5% significance level. This required 22 HO carriers and 22 non-carriers [26]. We changed the measure of the primary outcome from MAGE to CV after publication of the protocol but before data analysis, to adhere to the international consensus on using CV as the primary measure of glycaemic variability [31].

Analyses were performed as complete-case analyses since we had few missing observations and only for the outcomes. Furthermore, the few individuals with missing data had missing data on all measures of glucose variability and/or biochemical measures, making imputation inappropriate [34]. We excluded observations from participants who substituted three or more of the main meals during the three intervention days of a diet (i.e. breakfast, lunch and/or dinner) with foods having a different macronutrient composition. For glycaemic variability outcomes, we used data from each intervention period (i.e. two measurements [*j*] per person). We used a linear mixed model to assess the interaction effect between the diets (*d*; Greenlandic diet [reference] *d*=0 or Western diet *d*=1) and genotype (*g*; non-carriers *g*=0 or carriers *g*=1). We included all the matching variables (i.e. age [*a*], sex [*s*], BMI [*b*], Inuit ancestry [*i*; continuous, varying from 0 to 1] and study site/location [*l*; Maniitsoq or Nuuk]) as covariates as well as the random effect (*r*) of participant. Thus, the outcome for the *j*’th observation (1–2) on the *p*’th participant was modelled as:

$${Outcome}_{pj}= \alpha +\beta {d}_{pj}+\gamma {g}_{pj}+\delta {d}_{pj}{g}_{pj}+{\varepsilon a}_{pj}+ \theta {s}_{pj}+{\vartheta b}_{pj}+{\mu i}_{pj}+{\rho l}_{pj}+{r}_{p}+{e}_{pj}, {r}_{p}\sim N\left(0,{\tau }^{2}\right), {e}_{pj}\sim N(0,{\sigma }^{2})$$

For each outcome, we assessed whether model residuals were normally distributed, and no transformation was needed to meet the model assumptions. We tested the significance of the interaction effect using a likelihood ratio test.

For secondary outcomes, data were collected four times (*j*) (i.e. before and after each intervention period). Of these outcomes, we expected triacylglycerol levels to be most affected by the intervention. Hence, since there was no clinically relevant difference between the two baseline measurements of triacylglycerol, we categorised baseline measurements into one baseline category and similarly for the remaining secondary outcomes. We used a linear mixed model to assess the interaction effect between the diets (*d*; baseline [reference] *d*=0, Greenlandic diet *d*=1 or Western diet *d*=2) and genotype (*g*; non-carriers *g*=0 or carriers *g*=1) on the different outcomes. We included the same covariates as for the primary outcome as well as visit (*v*; 1, 2, 3 or 4), carry-over effect (*c*; 0, Western or Greenlandic) and the random effect (*r*) of participant. Thus, the outcome for the *j*’th observation (1–4) on the *p*’th participant was modelled as:

$${Outcome}_{pj}=\alpha +\beta {d}_{pj}+\gamma {g}_{pj}+\delta {d}_{pj}{g}_{pj}+{\varepsilon a}_{pj}+ \theta {s}_{pj}+{\vartheta b}_{pj}+{\mu i}_{pj}+{\rho l}_{pj}+{\tau v}_{pj}+ {\varphi c}_{pj}+{r}_{p}+{e}_{pj}, {r}_{p}\sim N(0,{\tau }^{2}){, e}_{pj}\sim N(0,{\sigma }^{2})$$

Data management and analyses were performed using R software version 4.3.0 [35].

## Results

**Fig. 1:**
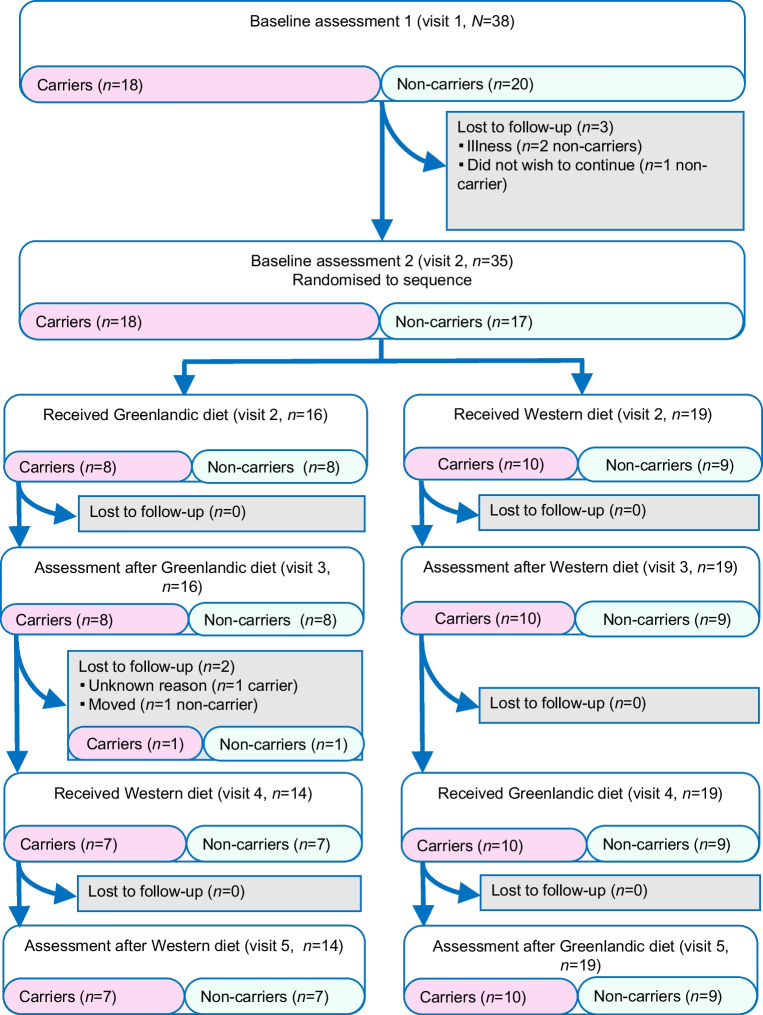
Flowchart of included participants

Thirty-eight participants, the majority of whom were female, were enrolled in the study (Table 1). Three non-carriers were lost to follow-up before randomisation. Of the remaining participants, eight carriers and eight non-carriers were allocated to receive the Greenlandic diet first, while ten carriers and nine non-carriers were allocated to receive the Western diet first (Fig. 1). One carrier and one non-carrier was lost to follow-up after visit 3 and we were only able to retrieve the continuous glucose monitoring (CGM) device for the non-carrier. In total, 17 carriers and 16 non-carriers completed both dietary interventions. One non-carrier was deemed non-compliant on day 2–3 of the Greenlandic diet (all meals had a considerable different macronutrient composition), hence, only day 1 was included. The CGM device of another non-carrier failed during wash-out. Hence, we had continuous glucose data on 17 carriers and 15 non-carriers for both diets. Blood samples after the Greenlandic diet were excluded for the non-compliant non-carrier.

**Table 1:** Baseline characteristics

Characteristic	Non-carriers (*N*=17)	Carriers (*N*=18)
Age	53.7 (46.3–58.8)	53.7 (36.7–58.6)
Female participants, *n* (%)	12 (70.6)	13 (72.2)
BMI, kg/m^2^	24.7 (21.2–27.7)	24.0 (21.4–25.9)
Waist-to-height ratio	0.56 (0.49–0.60)	0.49 (0.47–0.58)
Total cholesterol, mmol/l	5.30 (5.10–6.50)	5.90 (5.00–6.53)
HDL-cholesterol, mmol/l	1.61 (1.37–1.86)	1.69 (1.33–1.98)
LDL-cholesterol, mmol/l	3.20 (3.00–4.10)	3.50 (2.83–4.13)
VLDL-cholesterol, mmol/l	0.50 (0.50–0.60)	0.40 (0.30–0.60)
Non-HDL-cholesterol, mmol/l	3.77 (3.57–4.71)	4.13 (3.27–4.74)
Triacylglycerol, mmol/l	1.07 (1.01–1.38)	0.94 (0.71–1.31)
C-reactive protein, mg/l	0.95 (0.34–2.15)	0.66 (0.39–1.47)
Insulin, pmol/l	68.0 (42.0–81.0)	36.0 (27.3–52.0)
Glucose, mmol/l	5.70 (5.20–6.00)	5.55 (5.20–5.78)
HOMA-IR	2.91 (1.49–3.78)	1.44 (1.09–2.29)
HbA_1c_, mmol/mol	40.0 (38.0–41.0)	39.5 (37.3–43.0)
HbA_1c_, %	5.80 (5.60–5.90)	5.75 (5.53–6.10)
Inuit ancestry, %	73.0 (66.6–79.8)	74.1 (58.1–81.3)

Data are reported as median (IQR), except for sex distribution

All biochemical measures were measured in plasma, except for insulin and HbA_1c_, which were measured in serum

The groups were balanced in terms of the matching variables (i.e. sex, age, BMI and genetic admixture). Waist-to-height ratio, waist and hip circumference, plasma triacylglycerol, VLDL-cholesterol, glucose and C-reactive protein, serum insulin and HOMA-IR levels were numerically lower among carriers than non-carriers, while the levels of the remaining lipids were comparable (Table 1).

### Intervention effects on glucose variability and mean glucose

ESM Fig. 3 shows individual glucose levels, with more glucose peaks in non-carriers than carriers on the Western diet, while glucose levels appear similar and more stable in both groups on the Greenlandic diet.

**Fig. 2:**
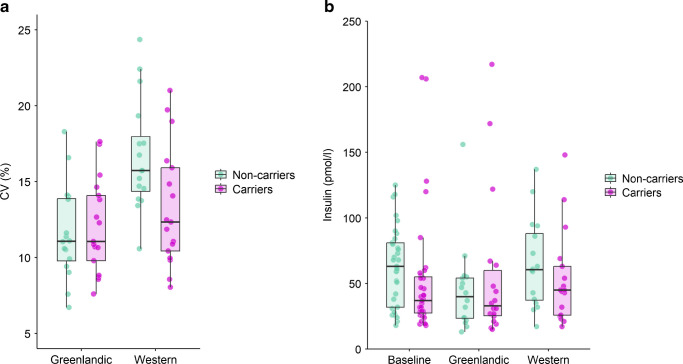
(**a**) CV in carriers and non-carriers during the two intervention diets. (**b**) Serum insulin in the two groups at the two baseline visits (pooled) and after each dietary intervention. The dots show raw data, the box shows the median and IQR, and the whiskers show the smallest and largest values within 1.5 × IQR from the hinges

Each of the three glycaemic variability outcomes (CV, MAGE and SD) were higher when non-carriers were on the Western diet compared with the Greenlandic diet (Table 2). However, they did not differ between diets in the carriers, and they were lower in carriers than in non-carriers only on the Western diet (see Table 2 for results of statistical analyses and Fig. 2a for raw CV data). Hence, there was a diet × genotype interaction effect on glycaemic variability. For example, CV was predicted to be 20% lower on the Western diet for a carrier compared with a similar non-carrier. Table 2 shows the effect of the Western diet compared with the Greenlandic diet for each group separately and the *p* value for testing the significance of the interaction effect. The Western diet was associated with higher mean glucose compared with the Greenlandic diet irrespective of carrier status.

**Table 2:** Glycaemic measures from glucose monitoring during three periods

Glycaemic outcome	Wash-out		Greenlandic diet		Western diet		Effect of diet and genotype (interaction)^a^		
	Non-carriers (*N*=17)	Carriers (*N*=17)	Non-carriers (*N*=16)	Carriers (*N*=17)	Non-carriers (*N*=16)	Carriers (*N*=17)	Effect of Western diet for non-carriers β (95% CI))	Effect of Western diet for carriers β + interaction effect δ (95% CI)	*p* value (interaction)
CV, %	15.9 (13.9–18.8)	15.3 (14.5–18.3)	11.1 (9.79–13.9)	11.1 (9.80–14.1)	15.7 (14.4–18.0)	12.4 (10.4–15.9)	5.23 (3.02, 7.45)	1.27 (−0.86, 3.40)	0.015
MAGE, mmol/l	2.24 (1.91–2.56)	2.05 (1.93–2.37)	1.37 (1.19–1.65)	1.57 (1.26–1.99)	2.26 (1.69–2.81)	1.77 (1.36–2.08)	0.84 (0.51, 1.17)	0.22 (−0.09, 0.53)	0.010
SD, mmol/l	0.906 (0.806–1.040)	0.838 (0.747–0.958)	0.586 (0.464–0.704)	0.581 (0.489–0.750)	0.876 (0.809–1.180)	0.713 (0.597–0.911)	0.39 (0.26, 0.51)	0.11 (−0.01, 0.24)	0.005
Mean glucose, mmol/l	5.46 (5.22–6.02)]	5.33 (5.18–5.76)	5.23 (4.85–5.76)	5.24 (4.98–5.39)	5.81 (5.56–6.09)	5.66 (5.34–6.12)	0.64 (0.39, 0.90)	0.37 (0.12, 0.61)	0.126
AUC, mmol/l × h	5.46 (5.22–6.02)	5.33 (5.18–5.77)	5.22 (4.85–5.76)	5.24 (4.98–5.39)	5.81 (5.56–6.11)	5.67 (5.34–6.11)	0.65 (0.39, 0.90)	0.37 (0.13, 0.61)	0.126

Data are reported as median (IQR)

The effect of the Western diet compared with the Greenlandic diet is reported for each genotype group (i.e. β[diet] and β[diet] + interaction [diet × genotype], respectively)

^a^Effects from a linear mixed model adjusted for age, sex, BMI, genetic admixture, study site and random effect of participant

### Intervention effects on circulating lipids, C-reactive protein and insulin

The Greenlandic diet increased the levels of HDL-cholesterol, LDL-cholesterol and total cholesterol and decreased the levels of triacylglycerol and VLDL-cholesterol compared with baseline (Table 3). In contrast, the Western diet increased the levels of triacylglycerol and VLDL-cholesterol and decreased the level of HDL-cholesterol, while no effect was seen on total cholesterol and LDL-cholesterol. The dietary effects on lipid levels did not differ between carriers and non-carriers.

**Table 3:** Plasma lipids, plasma C-reactive protein and serum insulin before and after each dietary intervention period

Variable	Before Greenlandic diet		After Greenlandic diet		Before Western diet		After Western diet		Difference^a^ (95% CI)		
	Non-carriers (*N*=17)	Carriers (*N*=18)	Non-carriers (*N*=16)	Carriers (*N*=18)	Non-carriers (*N*=16)	Carriers (*N*=17)	Non-carriers (*N*=16)	Carriers (*N*=17)	Non-carriers	Carriers	*p* value (interaction)
Total cholesterol, mmol/l	5.20 (4.70–5.60)	5.90 (4.85–6.20)	5.75 (5.28–6.20)	6.35 (5.25–6.80)	5.30 (4.98–5.75)	5.80 (5.00–6.40)	5.05 (4.60–5.85)	5.70 (4.50–6.20)	−0.53 (−0.82, −0.23)	−0.78 (−1.06, −0.5)	0.316
HDL-cholesterol, mmol/l	1.59 (1.38–1.87)	1.57 (1.33–1.99)	1.70 (1.51–1.91)	1.76 (1.53–2.14)	1.59 (1.38–1.88)	1.63 (1.42–1.87)	1.54 (1.37–1.75)	1.63 (1.25–1.86)	−0.23 (−0.32, −0.14)	−0.26 (−0.35, −0.17)	0.804
LDL-cholesterol, mmol/l	3.10 (2.80–3.30)	3.40 (2.93–3.88)	3.55 (3.28–4.03)	4.05 (3.33–4.48)	3.20 (2.78–3.58)	3.40 (3.00–4.10)	2.95 (2.45–3.70)	3.50 (2.60–3.70)	−0.57 (−0.84, −0.31)	−0.73 (−0.98, −0.47)	0.552
VLDL-cholesterol, mmol/l	0.500 (0.500–0.600)	0.400 (0.300–0.600)	0.400 (0.300–0.525)	0.400 (0.325–0.400)	0.500 (0.500–0.600)	0.400 (0.300–0.600)	0.700 (0.500–0.800)	0.500 (0.400–0.700)	0.25 (0.16, 0.34)	0.19 (0.10, 0.28)	0.285
Non-HDL-cholesterol, mmol/l	3.58 (3.39–3.89)	4.10 (3.32–4.61)	3.87 (3.64–4.62)	4.40 (3.72–4.95)	3.72 (3.27–4.31)	4.14 (3.31–4.55)	3.63 (3.07–4.37)	4.18 (3.15–4.47)	−0.3 (−0.56, −0.04)	−0.53 (−0.78, −0.27)	0.328
Triacylglycerol, mmol/l	1.190 (1.070–1.400)	0.935 (0.713–1.290)	0.900 (0.685–1.240)	0.910 (0.760–0.980)	1.090 (1.010–1.330)	0.920 (0.740–1.29)	1.570 (1.170–1.780)	1.110 (1.000–1.540)	0.61 (0.41,0.81)	0.43 (0.23, 0.62)	0.235
C-reactive protein, mg/l	0.950 (0.390–3.390)	0.705 (0.370–1.910)	0.760 (0.498–4.070)	0.955 (0.443–1.850)	1.470 (0.363–2.990)	0.570 (0.390–1.220)	0.830 (0.353–3.100)	0.450 (0.300–0.850)	−1.56 (−3.1, −0.01)	−0.26 (−1.76, 1.25)	0.234
Insulin, pmol/l	61.0 (38.0–80.0)	35.0 (25.8–54.0)	40.0 (23.5–54.3)	33.0 (25.5–60.0)	68.0 (30.3–83.3)	37.0 (29.0–58.0)	60.5 (37.3–88.0)	45.0 (26.0–63.0)	22.7 (5.05, 40.34)	−4.84 (−21.95, 12.27)	0.009

Data are reported as median (IQR)

^a^Effect of Western diet compared with the effect of Greenlandic diet (β[Western] − β[Greenlandic]) estimated with a linear mixed model including diet × genotype, sex, age, BMI, admixture, study site, visit and a random effect of participant

Insulin levels were not affected by the Western diet; however, insulin levels decreased considerably on the Greenlandic diet in non-carriers but were unaffected in carriers, hence, a genotype × diet interaction was observed (Table 3, Fig. 2b). For example, the insulin level was predicted to decrease by 28% in a non-carrier on the Greenlandic diet, while it was predicted to increase non-significantly by 5% in a corresponding carrier (55-year-old female participant with BMI 25 kg/m^2^ and 75% Inuit ancestry).

### Compliance

The two groups had comparable intakes of most of the intervention foods as well as other foods and beverages (ESM Figs 4, 5). Importantly, for the foods providing the greatest contribution to the sucrose intake, we observed similar intakes in the groups on the Western diet. The intake of whole grain bread was higher in HO carriers than in the non-carriers (ESM Fig. 4m). On the Greenlandic diet, participants consumed carbohydrate-containing foods that were not part of the intervention diet. The intake of foods such as juice and fruits was higher among carriers, while the intake of starchy carbohydrates was higher in non-carriers, though the groups only differed on day 1. Consumption of sucrose-containing drinks and starchy carbohydrates seemed slightly higher in the non-carriers on day 1 of the Greenlandic diet, though the groups did not differ significantly.

## Discussion

This study is the first to investigate dietary intervention in individuals with a complete loss of SI enzymatic function. Gene–diet interaction studies are often limited by a low allele frequency and/or impact; however, in this case the study was feasible in the small Indigenous population of Greenland due to the high frequency of the genetic variant causing the loss of function. It is furthermore unique in addressing a gene–diet interaction using an intervention study design. As the first clinical trial exploring SI as a potential treatment target for glucose regulation, we assessed the effect of a Western diet and a traditional Greenlandic diet in HO carriers and non-carriers of the *SI* loss-of-function variant and observed clear genotype differences on glucose homeostasis.

First, the glycaemic variability was higher in participants on the Western diet compared with the Greenlandic diet for non-carriers but not for the carriers. Hence, the dietary effect was modulated by the genotype, thus confirming our hypothesis of more stable blood glucose levels in carriers than in non-carriers on a Western diet. We expected this as a result of sucrose (and potentially starch) malabsorption among the carriers. We consider the difference of 20% in CV between the genotypes on the Western diet to be clinically relevant, considering the effect of existing pharmacological treatments for type 2 diabetes; in individuals with type 2 diabetes, vildagliptin reduced MAGE by 24.5% from baseline [36] and acarbose reduced CV by 26.3% [37]. We expect the effect of inhibiting SI to be larger in individuals with type 2 diabetes than in those without type 2 diabetes, like the participants included in this study, and furthermore the observed effect in this study might be underestimated rather than overestimated, as discussed later. Mean glucose was higher on the Western diet compared with the traditional Greenlandic diet, and mean glucose was non-significantly lower in carriers compared with non-carriers on the Western diet (*p*_interaction_=0.126), as expected from sucrose malabsorption. The results provide evidence that hindering SI enzymatic function can improve glycaemic management on a regular Western diet and thus potentially be a treatment target in individuals with diabetes [23, 25], or for prevention of type 2 diabetes in high-risk individuals [24]. If less energy is utilised on the Western diet for HO carriers compared with non-carriers, it could also be a potential weight-loss strategy. The Greenlandic diet led to lower glycaemic variability than the Western diet in non-carriers, suggesting better glycaemic management for the general population. However, with only 5 E% carbohydrates, the Greenlandic diet is likely difficult to follow and furthermore environmentally unsustainable outside the Arctic. Thus, although we do not recommend the Greenlandic diet as such for general diabetes prevention or management outside the Arctic, the study serves as a model for exploring genotype-specific (treatment target) effects in a Western society.

Second, the Greenlandic diet decreased fasting insulin levels in non-carriers but not in carriers of the *SI* loss-of-function variant. This decrease was parallel to the changes in CV and MAGE for non-carriers on the Greenlandic diet, likely as an expression of a normalisation in non-carriers of the hyperinsulinaemia associated with a modern Western diet with a high sucrose content [38], which is common in Greenland today [21]. In the study by Andersen et al homozygous carriers of the *SI* variant had non-significantly (*p*=0.088) lower insulin levels than other individuals [7], and our hypothesis of the Western diet being the cause of the phenotypic differences observed between HO carriers and non-carriers is therefore confirmed by the results of the present study.

No diet × genotype interactions were observed for lipids. However, the Western diet increased the levels of triacylglycerol and VLDL-cholesterol and decreased the level of HDL-cholesterol, while the Greenlandic diet had the opposite effect on these lipids, although it also increased LDL-cholesterol and total cholesterol levels. Some of the effects were likely mediated by the long-chain *n*-3 polyunsaturated fatty acids in the Greenlandic diet [39].

The Western intervention diet contained 11 E% sucrose, including naturally occurring sucrose, which is similar to the recommended maximum intake of added sugar, often sucrose, found in Western official guidelines (10 E%) [40, 41]. However, the sugar intake is higher in many individuals in Greenland and Western countries [16, 41, 42], hence we would expect the difference between HO carriers and non-carriers, and thereby the effect of inhibiting SI function, to be relatively larger in individuals consuming an average modern-day diet. Furthermore, we measured compliance without quantifying the intake in grams. If the groups differed in intake, carriers likely consumed less sucrose than the non-carriers, potentially underestimating the effect of the Western diet and the diet × genotype interaction. We cannot confirm that the diets were isoenergetic. However, we consider this likely since we provided all foods and meal instructions, and since the habitual energy intake was similar in the population surveys [7]. Blinding of the intervention was not possible, although we consider the risk of bias to be negligible since outcomes were objectively measured. We included both the female sex and male sex, supporting the generalisability of the findings across sexes. We did not formally assess potential effect modification by sex in the diet × genotype interaction analyses. However, the biological mechanisms underlying the observed effects are not expected to differ substantially between sexes.

SI accounts for nearly all sucrase and 60–80% of intestinal maltase activity [12], making it essential for digesting sugars and starches. Carriers of the *SI* loss-of-function variant likely cannot digest sucrose, as we observed no increase in blood glucose levels after a sucrose load, although it is not known whether other enzymes compensate for reduced maltase activity. Such compensation may not occur, or may take time to develop with pharmacological SI inhibition, potentially leading to stronger effects than those observed in carriers.

The study has some statistical limitations. It was underpowered compared with the sample size calculation and the power to detect diet × gene interaction effects is smaller than for main effects. However, it was difficult to obtain a meaningful sample size calculation due to lack of prior research. We still recruited a large number of both carriers and non-carriers, hence we consider the results useful. Expanding the recruitment in Greenland is logistically challenging due to small, dispersed settlements, which can only be reached by boat, aeroplane and/or helicopter (in good weather). We recommend future studies to screen for carriers over longer periods in Nuuk, Maniitsoq and/or East Greenland. Replication of the results using, for example, an observational design could also strengthen the conclusions. However, this was not feasible with the existing data at the current timepoint and was beyond the scope of the study. We did not adjust for multiple comparisons since the primary outcome was pre-specified and additional measures of glycaemic variability were included to examine consistency, making adjustment overly conservative.

Concerns regarding the accuracy of CGM devices has been raised [43] but these might be of less concern when using the devices for group comparisons in studies than for diabetes management. The Freestyle Libre Pro system used in this study has been found to have a relatively good accuracy in individuals with type 2 diabetes [44]. However, the accuracy of CGMs has also been found to depend on the blood glucose level, being lower in the hypoglycaemic range but acceptable for a wide range of glucose values, as for diabetes management [45].

This study supports SI inhibition as a potential therapeutic target but is limited by its short duration. Longer intervention periods are needed to assess effects on body composition, weight and cardiovascular disease, which are relevant considering the observational findings by Andersen et al [7]. Studying α-glucosidase inhibitors, which inhibit several carbohydrate-digesting enzymes including SI [46], could offer further insights (despite their unspecific action) by allowing large-scale studies. These drugs have also been linked to lower cancer risk, particularly gastrointestinal cancer [47]. Future work could also investigate the fate of undigested sucrose in carriers, including microbial fermentation and effects on intestinal permeability [48–50]. If beneficial effects are gained by colonic fermentation of undigested sucrose and short-chain fatty acid production, dose–response studies are relevant. Lastly, potential adverse effects of inhibiting SI should be addressed. While HO *SI* variant carriers in the Greenlandic population survey did not report more gastrointestinal symptoms than others [7], they reported gastrointestinal discomfort when consuming sucrose in a qualitative study [13]. The α-glucosidase inhibitors are generally not well tolerated due to diarrhoea and bloating when consuming carbohydrates [51]. However, an SI-specific inhibitor might cause fewer side effects. We did not record adverse events in the current study, being a limitation.

In conclusion, the results of this study show evidence of a diet × *SI* genotype interaction on glucose variability and fasting insulin levels, where a Western diet does not increase glucose variability in individuals with the loss-of-function *SI* variant in contrast to individuals with normal SI function. These findings show that SI inhibition could serve as a potential treatment target to improve glucose and insulin homeostasis and support further investigation for use in diabetes management. Moreover, the findings indicate that HO carriers in the Inuit population might cope better with the dietary westernisation that has occurred and is still occurring.

## Supplementary Information

Below is the link to the electronic supplementary material.

ESM (PDF 1719 KB)

## Data Availability

Requests for de-identified individual participant-level data can be submitted to Marit Eika Jørgensen, Steno Diabetes Center Greenland (maej@peqqik.gl). Data will not be made publicly available. The R scripts used for data management and statistical analyses are openly available at: https://github.com/NinnaSenftleber/SI_diet_intervention.git.

## Abbreviations

CGM: Continuous glucose monitoring
E%: Energy %
HO: Homozygous
MAGE: Mean amplitude of glucose excursion
SI: Sucrase-isomaltase
