# Supplementary material for: Dietary effect on glucose homeostasis is modulated by a loss-of-function variant in the sucrase-isomaltase gene: a randomised, dietary crossover intervention in Inuit
Source: Diabetologia. 2026 Apr 17;69(7):2015–28. doi: 10.1007/s00125-026-06723-4 (PMC13236788; doi:10.1007/s00125-026-06723-4)
Supplement: Supplementary file 1 — ESM (PDF 1719 KB) [file 125_2026_6723_MOESM1_ESM.pdf]

Electronic supplementary material

Contents

ESM Table 1: CONSIDER checklist: health research involving indigenous peoples. 2

ESM Fig. 1: The two study sites in Greenland 5

ESM Fig. 2: Overview of the study timeline and sampling. 6

ESM Fig. 3: Glucose levels (mmol/L) for each participant during the two intervention periods. 7

ESM Fig. 4A-P: Mean consumption of intervention foods and other foods/beverages during each day of the Western intervention diet. 8

ESM Fig. 5A-L: Mean consumption of intervention foods and other foods/beverages during each day of the Greenlandic intervention diet. 11

ESM Table 1: CONSIDER checklist: health research involving indigenous peoples.

ITEM		COMMENT	SECTION
Governance			
1.	Describe partnership agreements between the research institution and Indigenous-governing organization for the research, (e.g., Informal agreements through to MOU (Memorandum of Understanding) or MOA (Memorandum of Agreement)).	The study, including the user study, was planned and executed by researchers at both Greenlandic and Danish institutions, i.e., Steno Diabetes Center Greenland and Illisimatursarfik (University of Greenland), who were equally part of the project.	*-Contribution statement*<br>*- User study*
2.	Describe accountability and review mechanisms within the partnership agreement that addresses harm minimization.	The research project is based on agreements with the Greenlandic scientific ethical review system and collaboration agreements with the Greenlandic health care system, which aim to ensure that the research results benefit the population and public health in Greenland, and to ensure that the research itself and any resulting findings can be managed within the Greenlandic health care system.	
3.	Specify how the research partnership agreement includes protection of Indigenous intellectual property and knowledge arising from the research, including financial and intellectual benefits generated (e.g., development of traditional medicines for commercial purposes or supporting the Indigenous community to develop commercialization proposals generated from the research).	The research project does not appear to have any commercial potential.	
Prioritization			
4.	Explain how the research aims emerged from priorities identified by either Indigenous stakeholders, governing bodies, funders, non-government organization(s), stakeholders, consumers, and empirical evidence	The study emerged from published research	*Introduction*
Relationships (Indigenous stakeholders/participants and Research team)			
5.	Specify measures that adhere and honor Indigenous ethical guidelines, processes, and approvals for all relevant Indigenous stakeholders, recognizing that multiple Indigenous partners may be involved, e.g., Indigenous ethics committee approval, regional/national ethics approval processes.	The study received approval from the Ethics Committee of Greenland	*Methods*
6.	Report how Indigenous stakeholders were involved in the research processes (i.e., research design, funding, implementation, analysis, dissemination/recruitment).	- Research design and acceptability of study: user study was conducted prior to the intervention carried out by colleagues at Illisimatursarfik (University of Greenland).<br>- Funding: Pinngortitaleriffik (Greenland Institute of Natural Resources), Greenland Research Council, and Brugseni.<br>- Implementation/recruitment: two Indigenous and local research assistants recruited participants, conducted interviews, and managed overall participant communication.<br>- Dissemination: to the general population in Greenland. We plan to communicate findings directly to participants when we have more results ready (we collected data for other purposes as well, e.g. food preferences).	*- User study*<br>*- Authors' relationships and activities*<br>*- Participants and recruitment*<br>*- Acknowledgements*

7.	Describe the expertise of the research team in Indigenous health and research.	The research project is locally based in Greenland, where MEJ and MMBC have experience as health care professionals within the Greenlandic health care system. NS, MMBC, BC, FFS, TH, and MEJ have long-standing experience with research in Inuit health.	
Methodologies			
8.	Describe the methodological approach of the research including a rationale of methods used and implication for Indigenous stakeholders, e.g., privacy and confidentiality (individual and collective)	The research was anchored at Steno Diabetes Center Greenland and the prior user study was carried out by an Indigenous researcher at Illisimatursarfik (University of Greenland). Two Indigenous and local research assistants were part of the research team collecting data.	*Methods*
9.	Describe how the research methodology incorporated consideration of the physical, social, economic and cultural environment of the participants and prospective participants. (e.g., impacts of colonization, racism, and social justice). As well as Indigenous worldviews.	A user study was carried out before the intervention start to ensure both that the intervention would be acceptable both in terms of possible side effects among carriers as well as practically. Furthermore, the study addressed opinions towards the research topic and whether the individuals considered it relevant and acceptable to study.	*User study*
Participation			
10.	Specify how individual and collective consent was sought to conduct future analysis on collected samples and data (e.g., additional secondary analyses; third-parties accessing samples (genetic, tissue, blood) for further analyses).	Informed consent from participants. Participants who have not previously been whole genome sequenced further gave informed consent for this.	*Methods*
11.	Described how the resource demands (current and future) placed on Indigenous participants and communities involved in the research were identified and agreed upon including any resourcing for participation, knowledge, and expertise	Participants received the intervention foods for free, which covered the dietary requirements for the whole household.	*Intervention*
12.	Specify how biological tissue and other samples including data were stored, explaining the processes of removal from traditional lands, if done, and of disposal.	Blood samples have been stored in Greenland until data collection was complete, and then shipped to Denmark for analysis, where they are stored until analyses are complete and destroyed afterwards. Blood samples for potential figure analyses are stored in Nuuk, Greenland. Details regarding sampling and shipping are described in the published protocol	*Blood samples after an overnight fast*
Capacity			
13.	Explain how the research supported the development and maintenance of Indigenous research capacity (e.g., specific funding of Indigenous researchers).	Employment and training of the two Indigenous research assistants.<br>Research dissemination to the general population, including school kids. Part of one of the research grants (from the Greenland Research Council) covered costs in the user study.	- *Participants and recruitment*<br>- *Acknowledgements*
14.	Discuss how the research team undertook professional development opportunities to develop the capacity to partner with Indigenous stakeholders?	See point 8.	
Analysis and interpretation			
15.	Specify how the research analysis and reporting supported critical inquiry and a strength-based approach that was inclusive of Indigenous values.	Participants of the user study prior to the intervention supported the research project and a previous sharing circle study in Greenland also	*User study*

		showed that the participants supported genetic research in Greenland.	
Dissemination			
16.	Describe the dissemination of the research findings to relevant Indigenous governing bodies and peoples.	Findings and knowledge on the Arctic-specific condition has been communicated to the general Greenlandic population.	*Acknowledgements*
17.	Discuss the process for knowledge translation and implementation to support Indigenous advancement (e.g., research capacity, policy, investment).	Not discussed at this point.	

ESM Fig. 1: The two study sites in Greenland

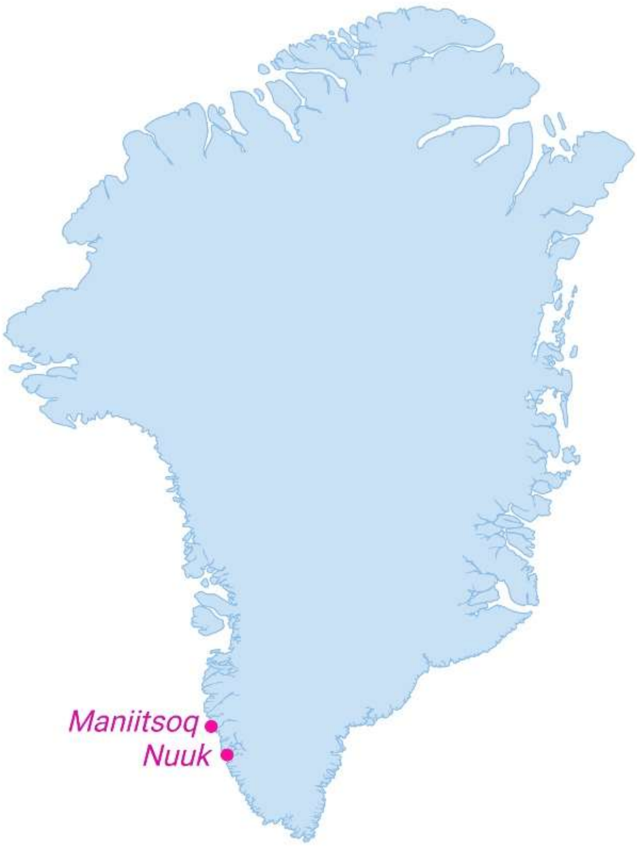

ESM Fig. 2: Overview of the study timeline and sampling.
AUC, area under the curve; CGM, continuous glucose monitoring; CRP, C-reactive protein; CV, coefficient of variation; HDL-C, HDL-cholesterol; LDL-C, LDL-cholesterol; MAGE, mean amplitude of glucose excursion; non-HDL-C; non-HDL-cholesterol; VLDL-C, VLDL-cholesterol.

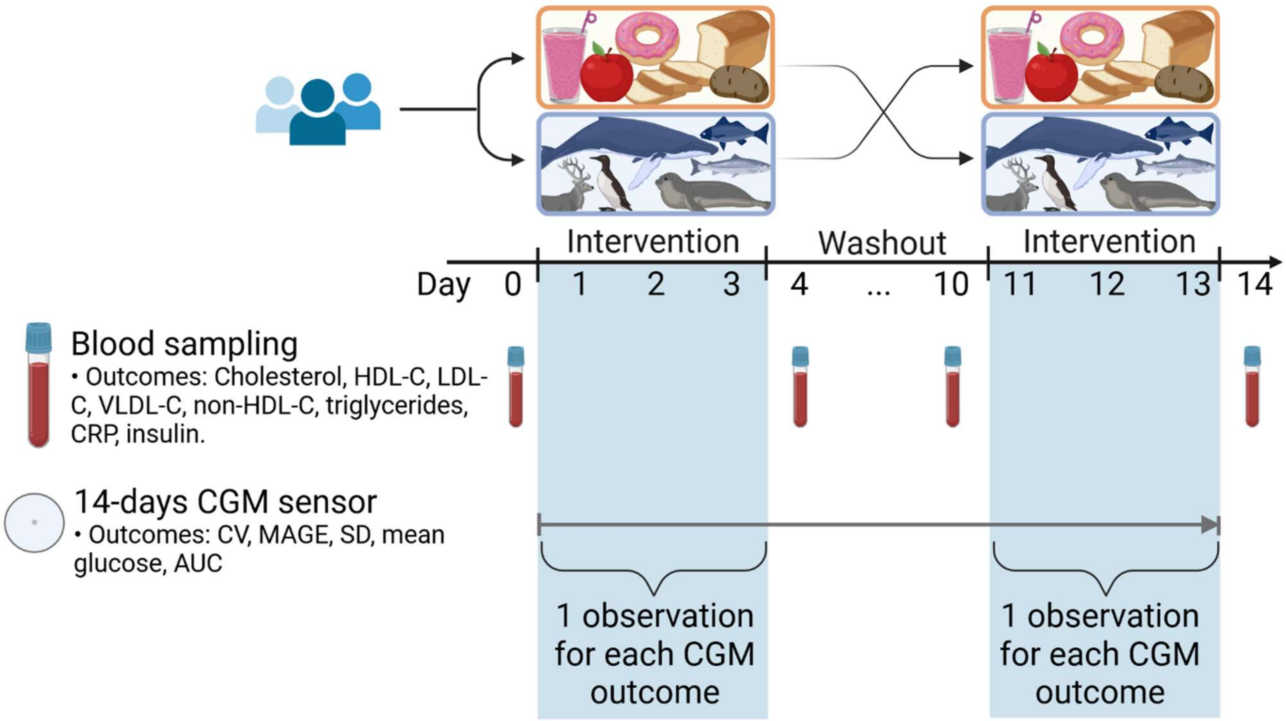

ESM Fig. 3: Glucose levels (mmol/L) for each participant during the two intervention periods.

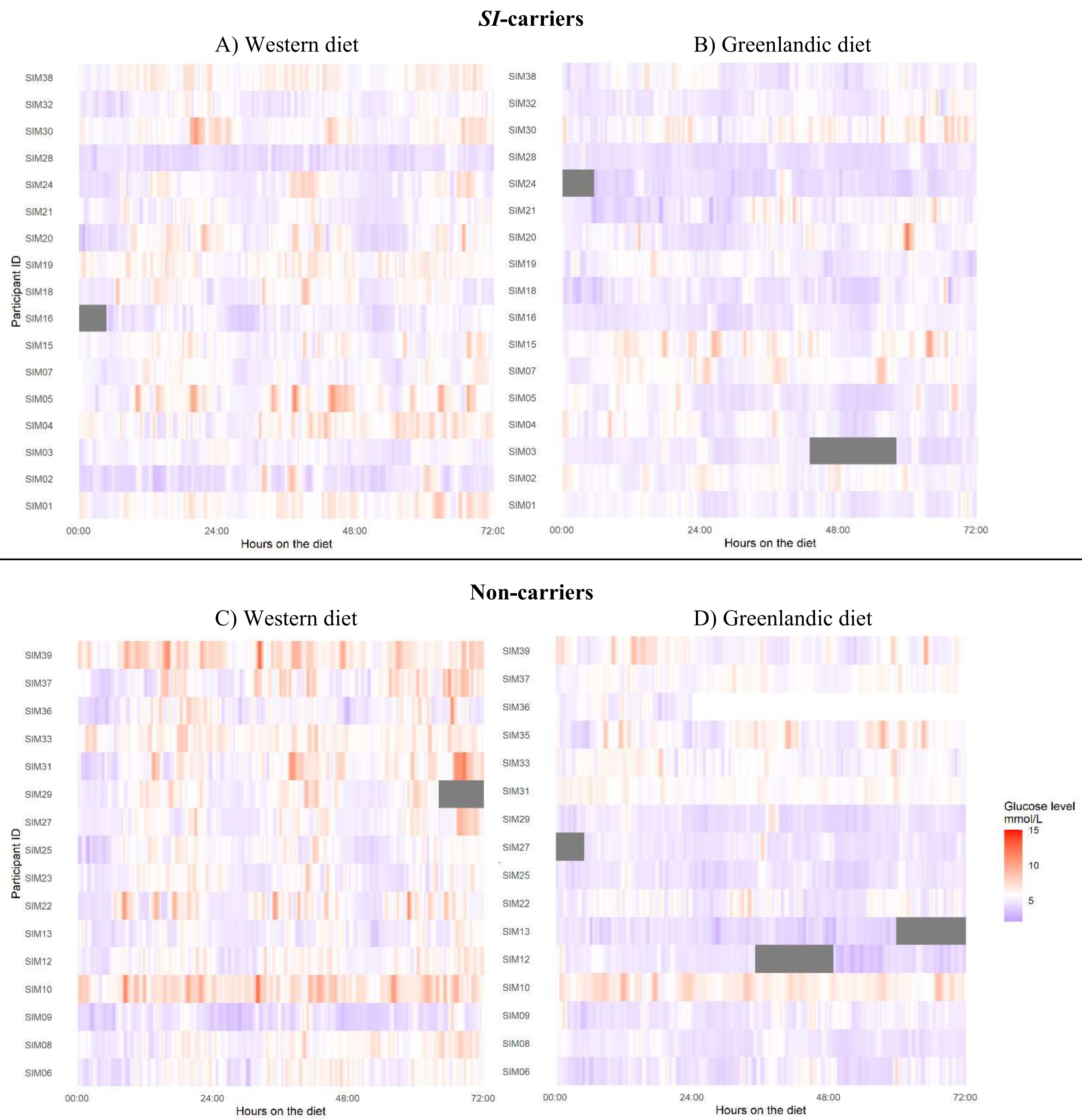

A, C: Three days of Western intervention diet. B, D: Three days of Greenlandic intervention diet. Grey areas show missing data.

ESM Fig. 4A-P: Mean consumption of intervention foods and other foods/beverages during each day of the Western intervention diet.

A)

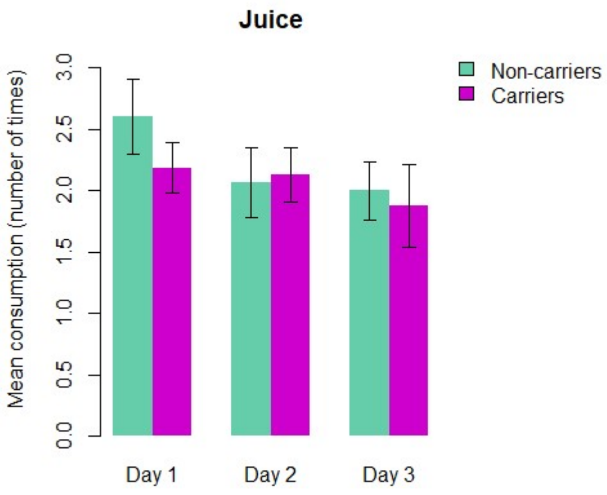

B)

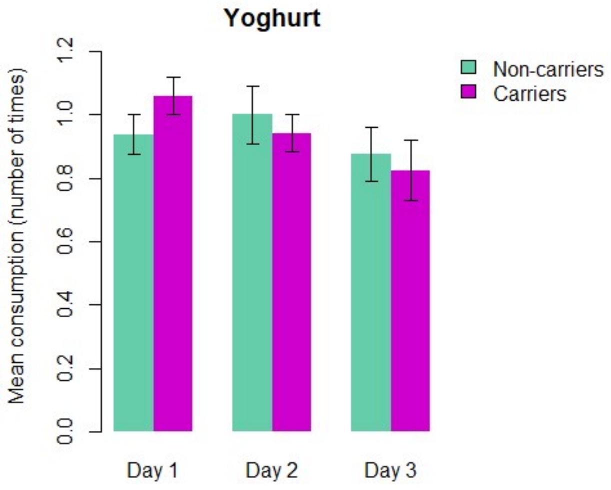

C)

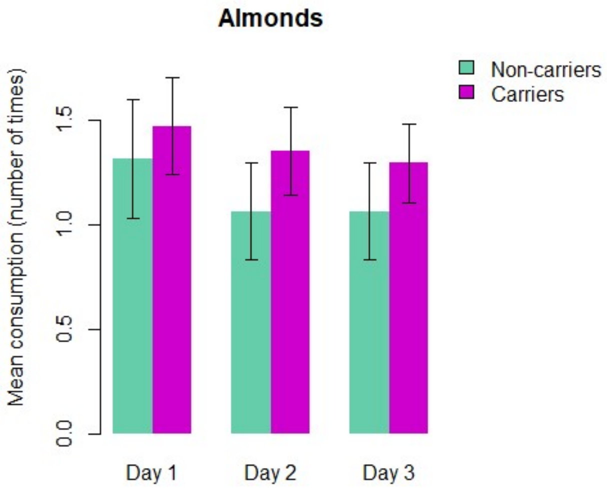

D)

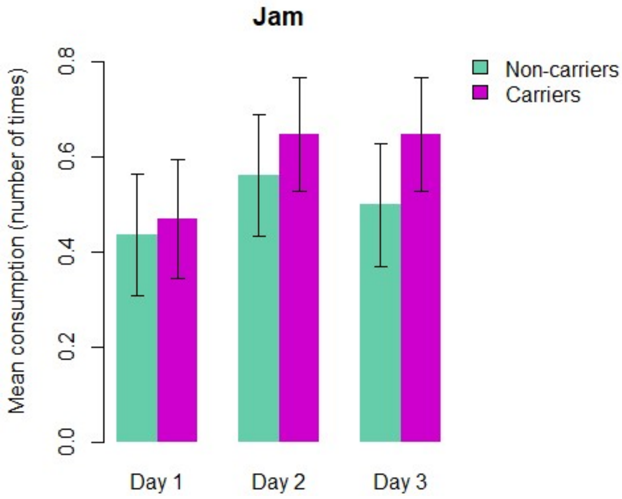

E)

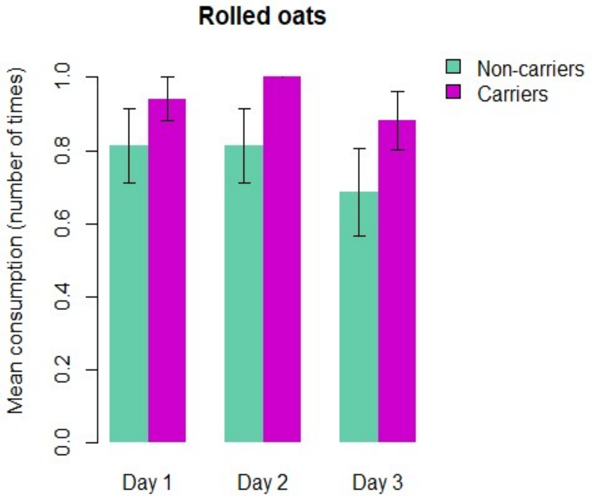

F)

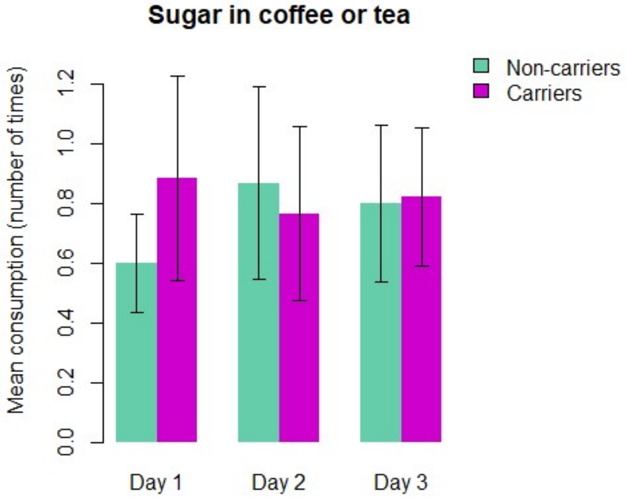

G)

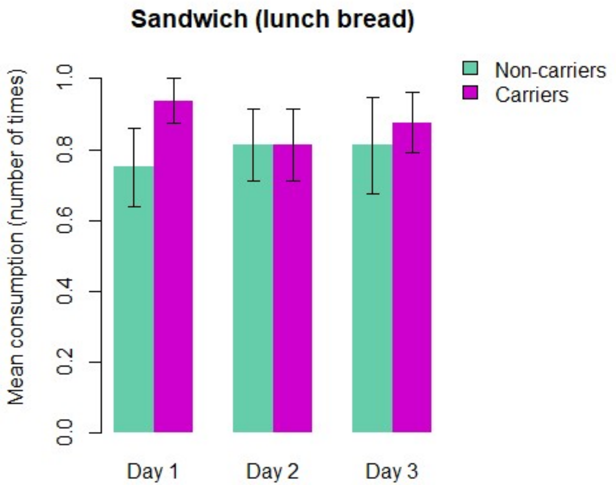
Sandwich (lunch bread)
Mean consumption (number of times)
0.0
0.2
0.4
0.6
0.8
1.0
Day 1
Day 2
Day 3
Non-carriers
Carriers

H)

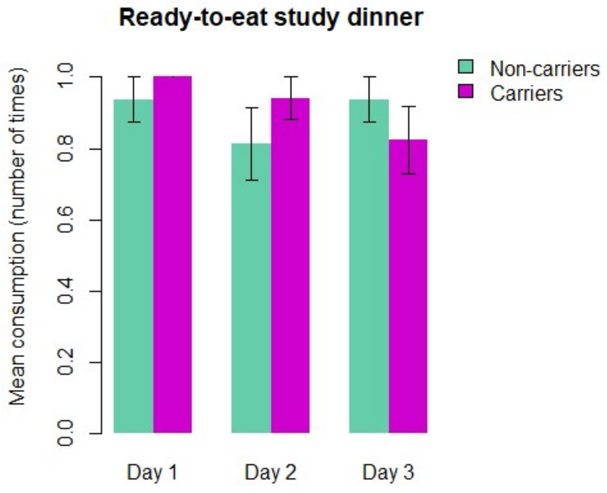
Ready-to-eat study dinner
Mean consumption (number of times)
0.0
0.2
0.4
0.6
0.8
1.0
Day 1
Day 2
Day 3
Non-carriers
Carriers

I)

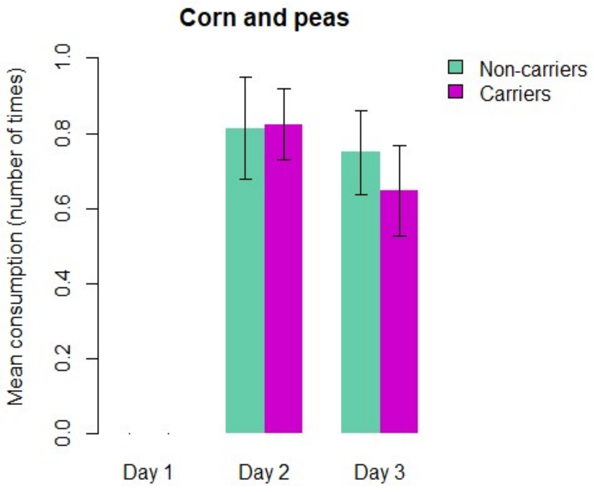
Corn and peas
Mean consumption (number of times)
0.0
0.2
0.4
0.6
0.8
1.0
Day 1
Day 2
Day 3
Non-carriers
Carriers

J)

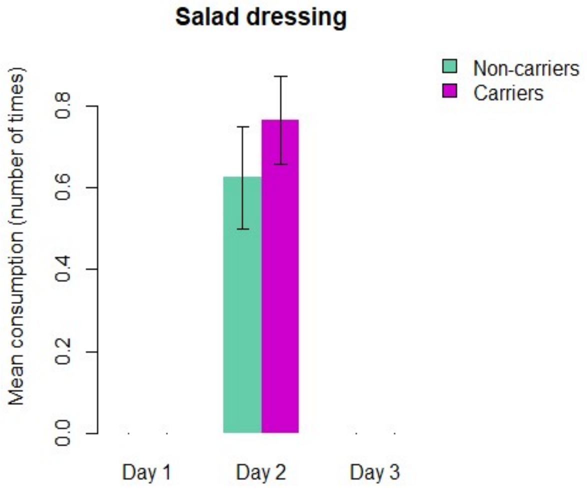
Salad dressing
Mean consumption (number of times)
0.0
0.2
0.4
0.6
0.8
Day 1
Day 2
Day 3
Non-carriers
Carriers

K)

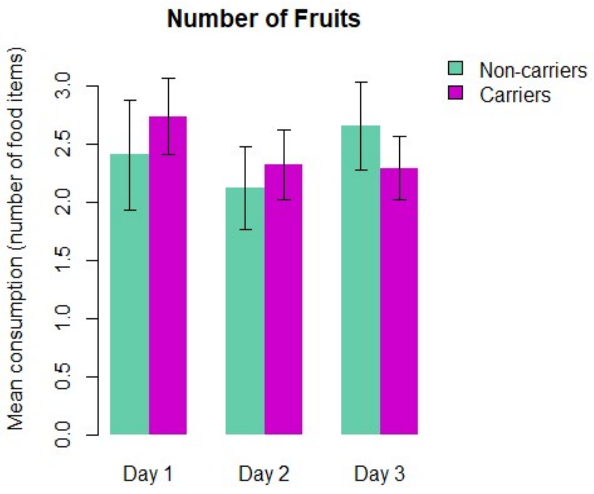
Number of Fruits
Mean consumption (number of food items)
0.0
0.5
1.0
1.5
2.0
2.5
3.0
Day 1
Day 2
Day 3
Non-carriers
Carriers

L)

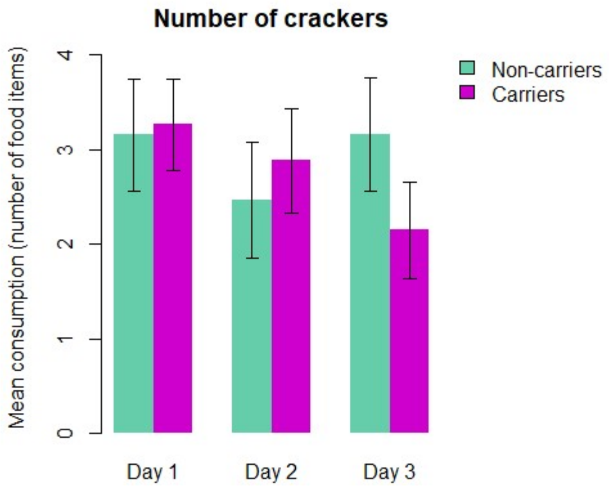
Number of crackers
Mean consumption (number of food items)
0
1
2
3
4
Day 1
Day 2
Day 3
Non-carriers
Carriers

M)

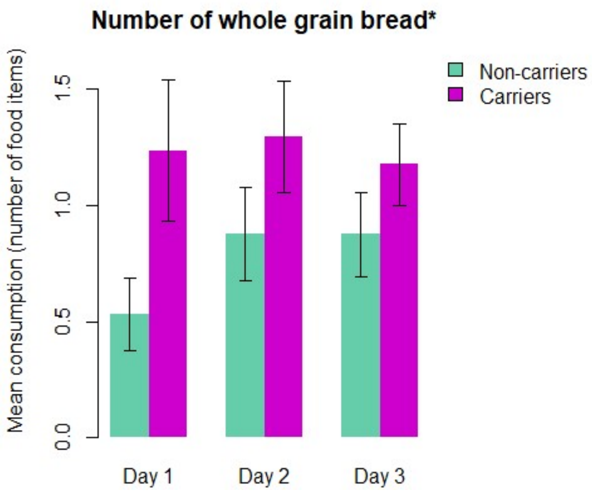

N)

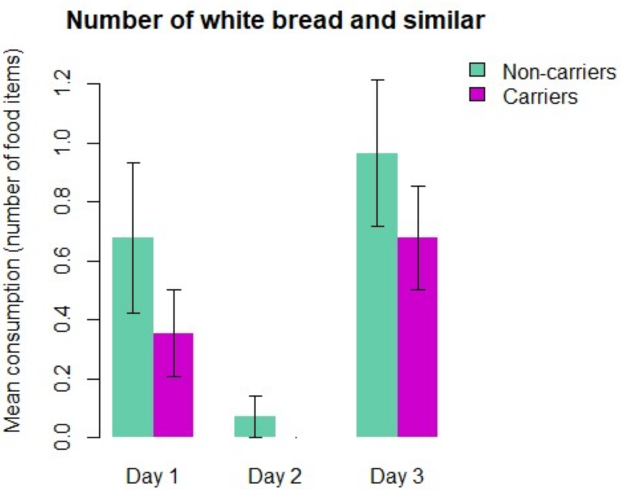

O)

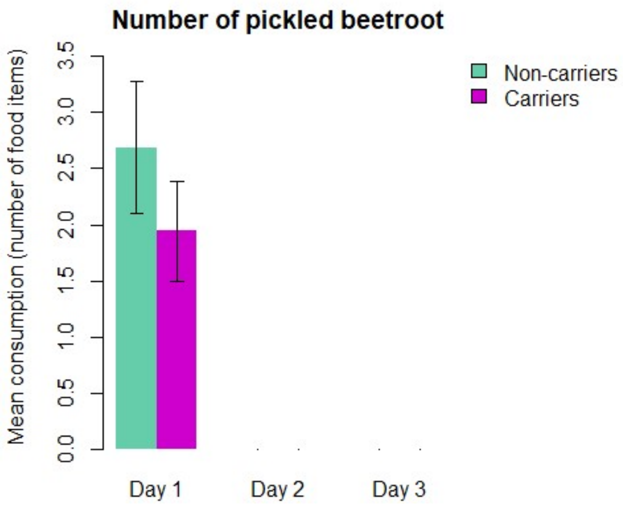

P)

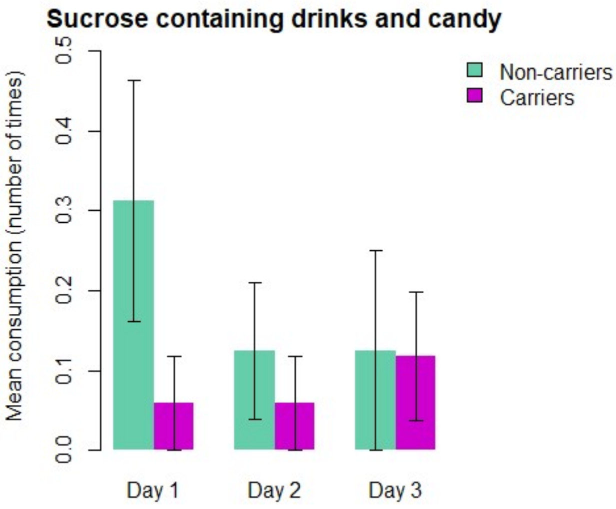

Intake given as frequency (number of times) for all foods except for K)-O). Data presented as means with standard errors. *Significant (p<0.05) difference between carriers and non-carriers in overall intake based on Poisson regression of intake, including genotype (0, 1) and day (1, 2, 3) as covariates.

ESM Fig. 5A-L: Mean consumption of intervention foods and other foods/beverages during each day of the Greenlandic intervention diet.

A)

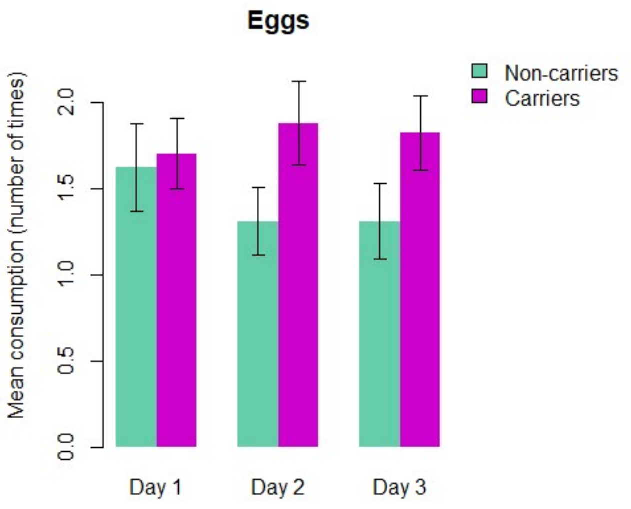

B)

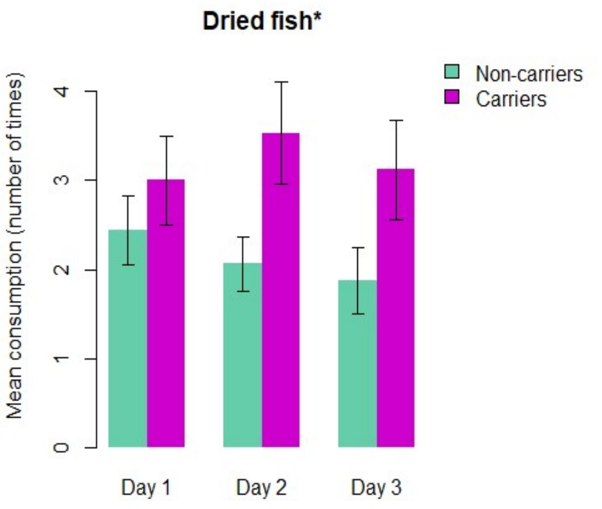

C)

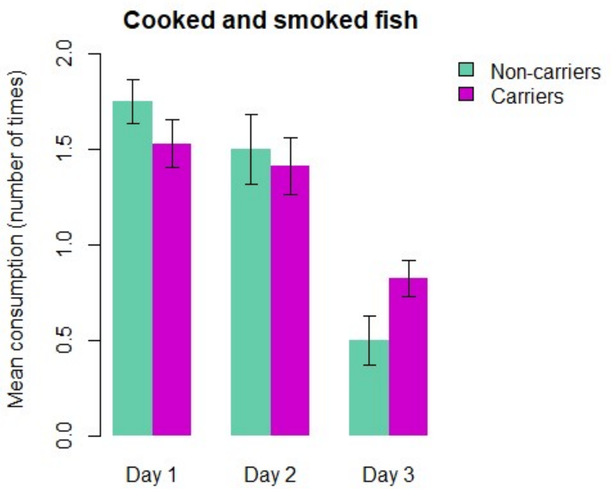

D)

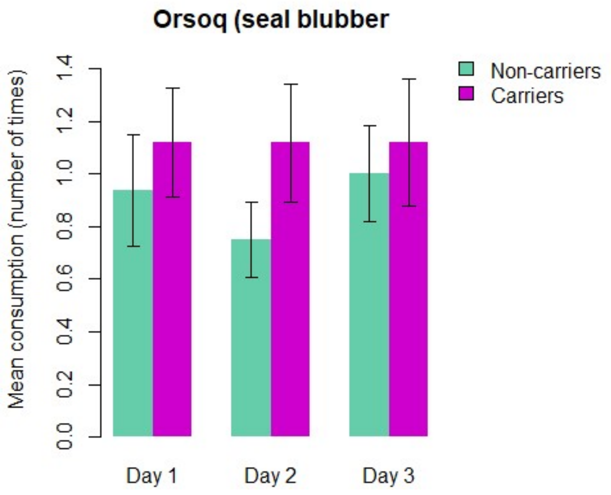

E)

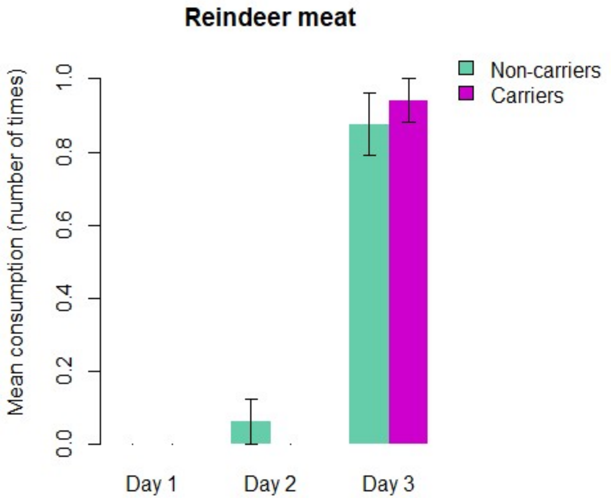

F)

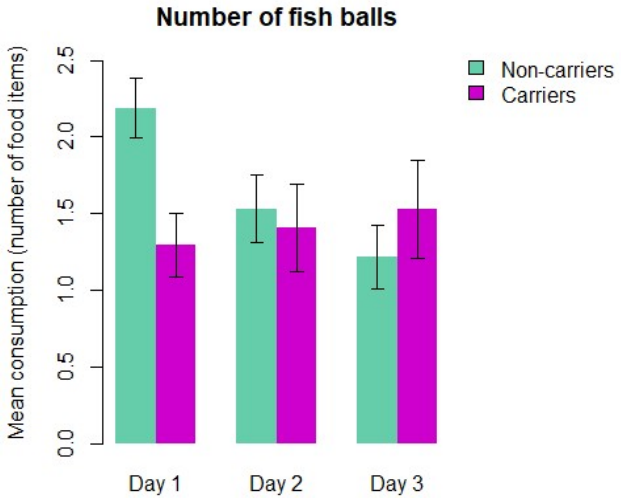

G)

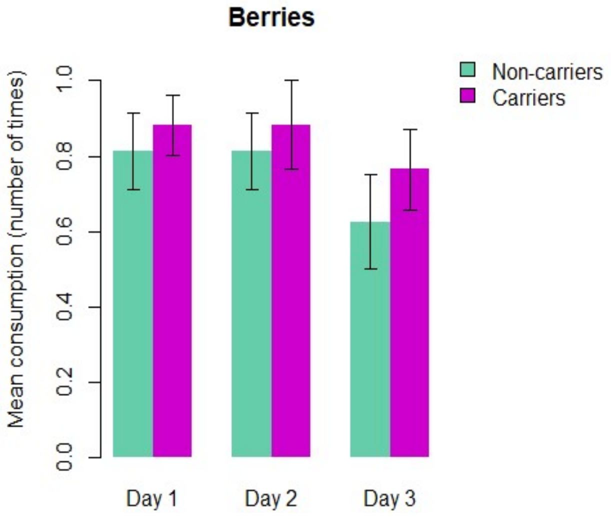

H)

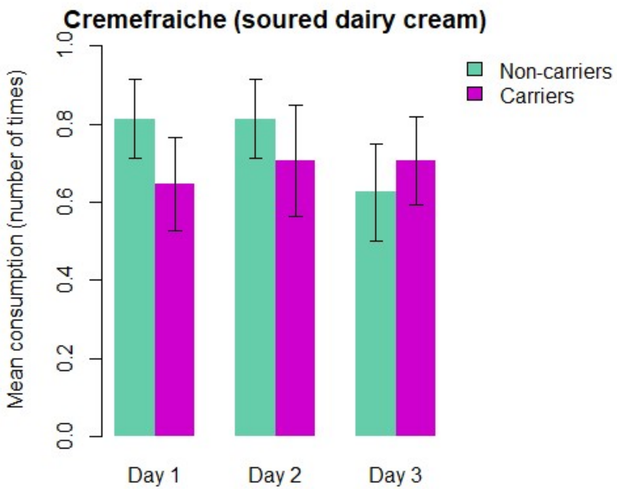

I)

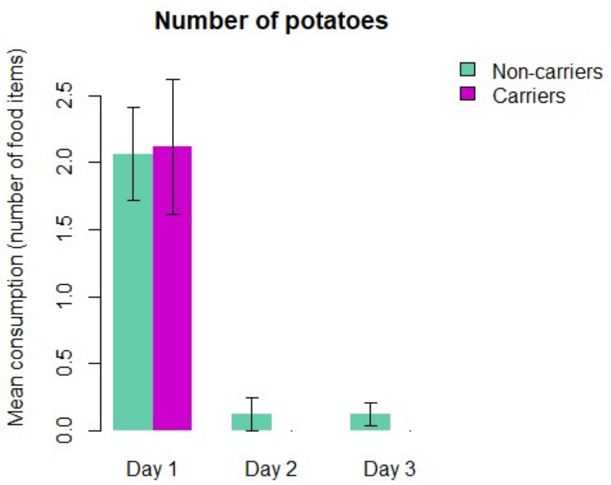

J)

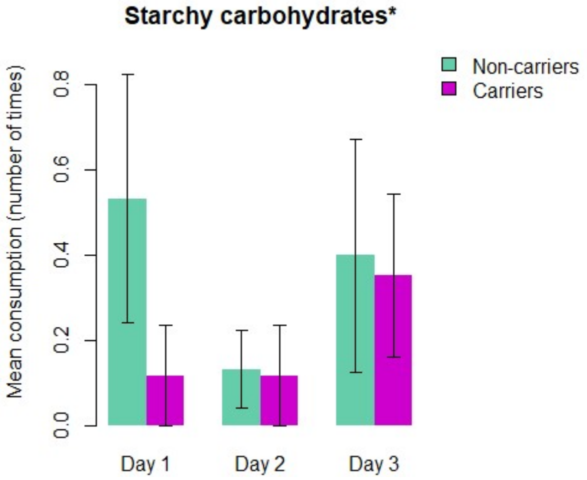

K)

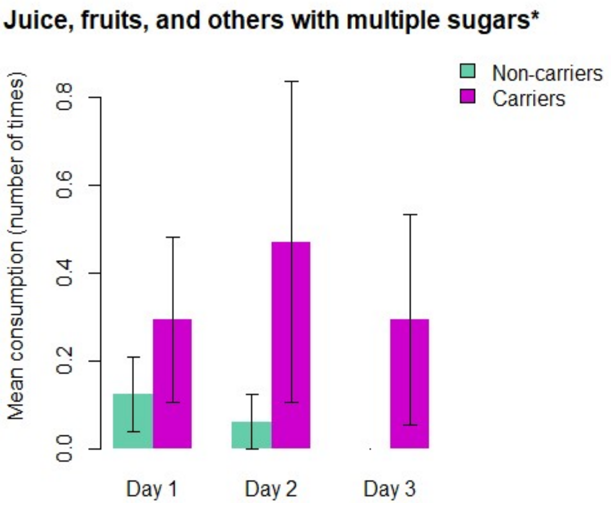

L)

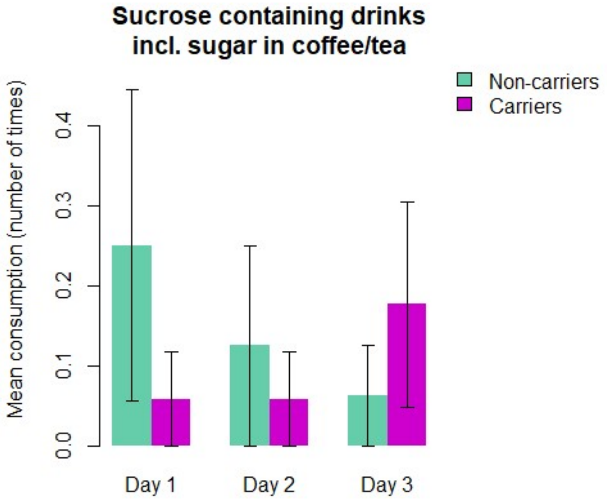

Intake given as frequency (number of times) for all foods except for F) and I). As in the statistical analyses of the outcomes, data from day 2-3 have been removed for the non-carrier, that was non-compliant on these days. Data presented as means with standard errors. *Significant ($p<0.05$) difference between carriers and non-carriers in overall intake based on Poisson regression of intake, including genotype (0, 1) and day (1, 2, 3) as covariates.
